# Delayed massive hemothorax after blunt thoracic trauma requiring thoracotomy by VATS: a case report

**DOI:** 10.1093/jscr/rjab537

**Published:** 2022-01-17

**Authors:** Chan Hee Park, Kyeong Eui Kim, Min Cheol Chae, Jeong Woo Lee

**Affiliations:** Department of Surgery, School of Medicine, Keimyung University and Dongsan Medical Center, Daegu, Korea; Department of Surgery, School of Medicine, Keimyung University and Dongsan Medical Center, Daegu, Korea; Department of Chest Surgery, School of Medicine, Keimyung University and Dongsan Medical Center, Daegu, Korea; Department of Surgery, School of Medicine, Keimyung University and Dongsan Medical Center, Daegu, Korea

## Abstract

Delayed hemothorax after thoracic trauma is a rare type of thoracic injury that may require angioembolization and surgical treatment. We report a case of a 59-year-old man with a delayed massive hemothorax from a fall-induced blunt thoracic trauma, causing multiple right lower rib fractures. The patient fell from a chair while standing on and working from it. He was diagnosed with right 7th–11th rib fractures, scanty hemothorax and liver contusion. The patient was hospitalized and received conservative treatment, and a delayed massive hemothorax was present on the fourth day after the injury. Chest tube drainage and video-assisted thoracoscopic surgery were performed, and the patient was discharged 16 days after the surgery without bleeding or other complications. Delayed hemothorax should be considered when thoracic trauma patients complain of chest discomfort, dyspnea, cold sweating or fatigue. Early recognition, appropriate diagnosis and rapid intervention can improve prognosis and lead to successful patient treatment.

## INTRODUCTION

Thoracic trauma accounts for ~10–15% of all traumas, and 25% of these cases may lead to death. Delayed massive hemothorax caused by blunt thoracic trauma is rare [1, 2]. Massive hemothorax is defined as blood drainage >1500 ml after a closed thoracostomy or continuous bleeding at 200 ml/h for at least 4 h [2]. Blunt thoracic trauma accounts for 70% of all thoracic traumas, and some instances may require angioembolization and surgical treatment. These rare topics are reviewed in this report. Our case report was approved by the Institutional Review Board (approval number: DSMC 2021-09-015).

## CASE REPORT

**Figure 1:**
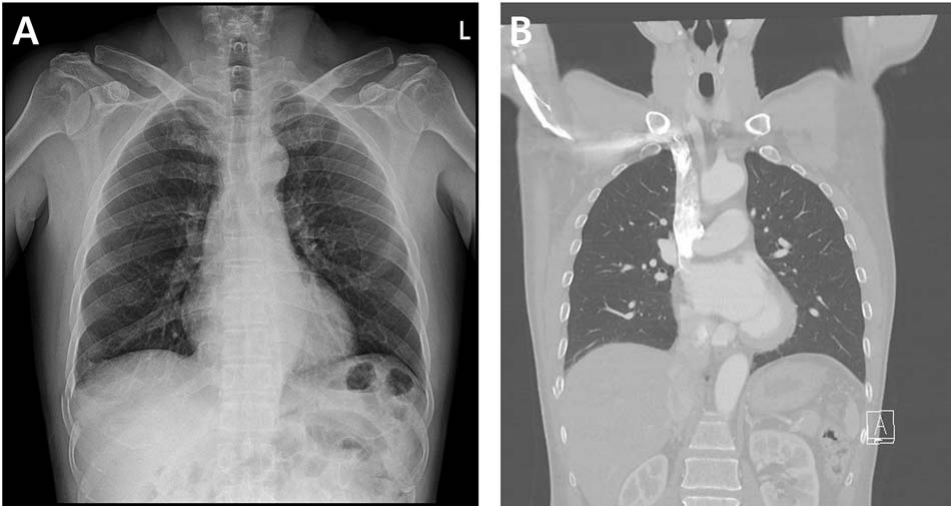
Radiographic findings. (**A**) Initial chest X-ray showed no evidence of hemothorax. (**B**) Initial chest CT also showed no evidence of hemothorax.

**Figure 2:**
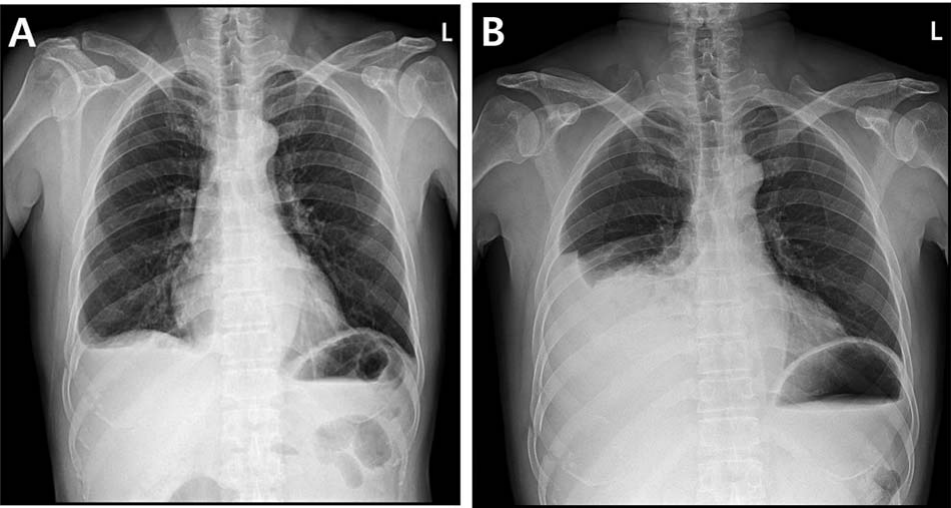
Radiographic findings. (**A**) Day of symptoms, chest X-ray in the morning. (**B**) Chest X-ray after symptoms develop.

**Figure 3:**
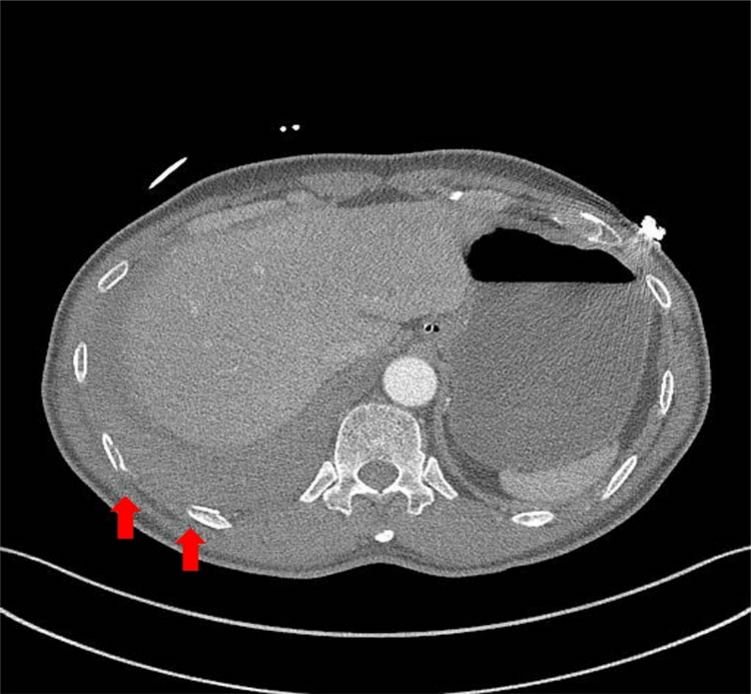
Chest contrast-enhanced CT showed massive hemothorax with multiple rib fractures including 10th, 11th ribs (arrow) with no active extravasation of contrast.

**Figure 4:**
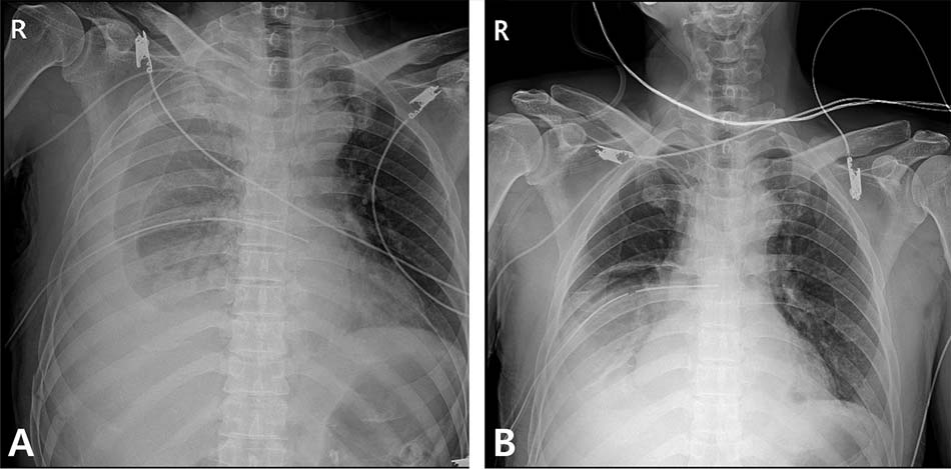
Radiographic findings. (**A**) After chest tube insertion. (**B**) 12 h after chest tube insertion.

**Figure 5:**
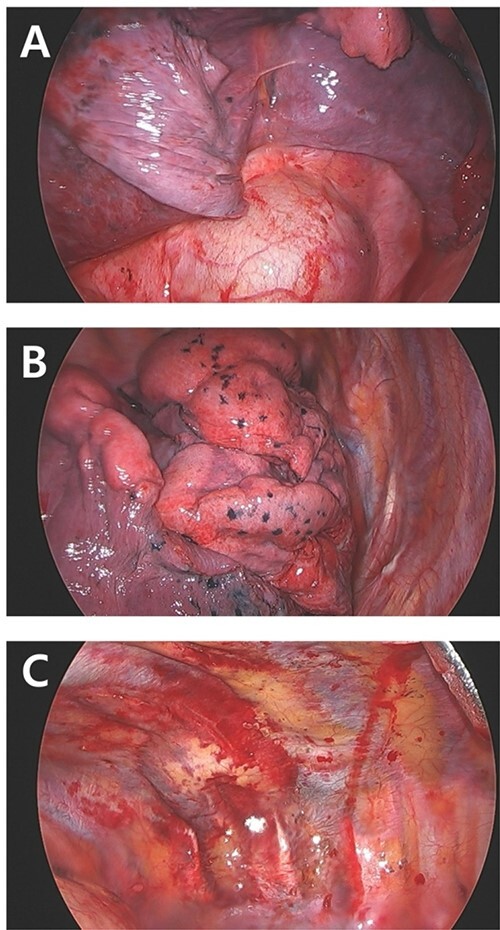
Intraoperative finding. (**A**) No active bleeding focus on right diaphragm. (**B**) Lung contusion but no bleeding. (**C**) Pleural laceration due to fractured and displaced 10th rib (arrow).

**Figure 6:**
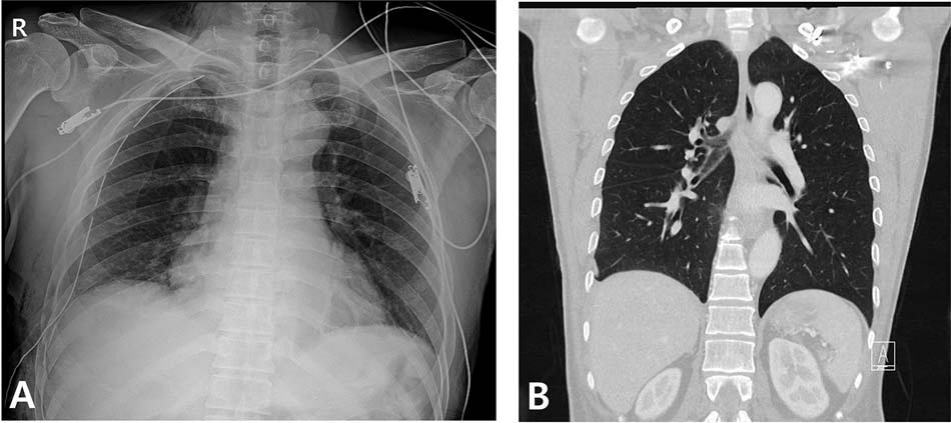
Radiography and CT scan findings. (**A**) Immediate postoperative chest radiography. (**B**) A month after surgery, outpatient chestCT.

A 59-year-old man was admitted with multiple rib fractures and liver contusion due to a fall injury. He was standing on a chair for working at a farm, and it was knocked over causing him to fall and hit his flank against the corner of the chair. There was continuous pain while resting at the right flank and severe pain with pressure was noted, but no external wounds or bruises were observed. There were right 7th–11th rib fractures, scanty pneumothorax, minimal hemothorax and a 2-cm-sized liver contusion in abdomen and chest computed tomography (CT) scan (Fig. 1A, B). He was hospitalized for pain control and close observation in the general ward, and conservative management was initiated. Also, no significant changes were noted in the following daily follow-up chest radiographs. The patient suddenly complained of right-sided chest and back pain aggravation, cold sweating and fatigue 80 h after the traumatic injury. His mental status was alert, but v/s including systolic blood pressure (SBP) of 100–120 mmHg, heart rate (HR) of 40-60 beats/min and oxygen saturation of 100% during the admission changed to an SBP of 86/60 mmHg, HR of 88 beats/min and oxygen saturation of 97% when the symptoms occurred. Chest radiography was performed after the patient experienced aggravated symptoms, such as right-sided flank pain, cold sweating and fatigue. Compared to the previous scans, signs of increased opacification and peribronchial and parenchymal infiltrations were observed, which were indicative of hemothorax (Fig. 2A, B). We performed enhanced dynamic chest CT to identify any presence of active bleeding. On the chest CT scan, a large amount of hemothorax was identified in the right lung field along with multiple fractures of the right ribs. However, there were no signs of contrast leakage indicative of active bleeding (Fig. 3). Hemoglobin levels decreased from 13.1 g/dl on the day before the symptoms appeared to 11.5 g/dl at the onset of symptoms and to 9.4 g/dl after 2 h. Four packs of RBC transfusion and fluid were administered to the patient, and he was moved to the intensive care unit for close monitoring. And tube thoracostomy was performed, and 1600 ml of fresh blood was drained (Fig. 4A). The following day, 500 ml of blood was drained through the chest tube, but his v/s were stable (Fig. 4B). The amount of bleeding through the chest tube was decreased, but the drained fluid was fresh blood. And we thought that the remaining hematoma was not effectively drained, so the patient’s respiratory discomfort could persist and cause uneffective ventilation. And then we consulted with the Department of Thoracic Surgery, and video-assisted thoracoscopic surgery (VATS) exploratory thoracotomy was performed to identify the bleeding source caused by displaced rib and evacuate the large amount of hematoma. There was a large volume of hematoma within the pleural space and between the right lower lobe, diaphragm and fissure, but no active bleeding point was located. The fractured right 10th rib pierced through the pleural space and was displaced to the thoracic cavity, which was easily reduced. There was no injury surrounding the diaphragm, and although the general lung and chest wall contusions were severe, there were no signs of lung parenchymal lacerations (Fig. 5A–C). Chest tube drainage was serous, and there was no further bleeding. There were no signs of bleeding or any other abnormal findings on chest CT performed at the outpatient clinic, and the patient had no complaints of any symptoms (Fig. 6A, B).

## DISCUSSION

Delayed hemothorax is a rare complication of thoracic trauma. According to one study, delayed hemothorax was seen in 12.3% of patients with minor thoracic injury within 14 days of the injury, and only a small number of patients required surgery [1, 3]. Other studies have reported the onset of delayed hemothorax from a few hours to 11 days after the injury. Common causes of massive hemothorax after thoracic trauma are injuries of the intercostal artery, laceration of the lung and diaphragmatic injury [4]. However, in this case study, a delayed hemothorax occurred even without the injuries mentioned above.

Risk factors that can lead to delayed hemothorax post-thoracic blunt trauma include the location of the fracture and the number of fractures identified on images [5, 6]. Furthermore, a cohort study by Chien et al. [6] reported that the number of rib fractures is an important predictor of pulmonary complications, with three or more fractures being the most sensitive risk factor.

It is well known that the amount of bleeding should be checked first, and then deciding whether surgical or other interventional procedures are necessary. If the patient is hemodynamically stable and there is no active bleeding, blood drainage from the pleural space with tube thoracostomy should be performed first. Then, the status of the drainage must be checked along with conservative treatments [3, 4, 7, 8]. In the case of continuous active bleeding or unstable v/s, embolization through angiography or surgical treatment such as open thoracotomy or VATS must be considered. The purpose of surgery for delayed hemothorax is to control bleeding within the thoracic cavity and hematoma evacuation. Compared to traditional open thoracotomy, VATS has been reported to have practicality and stability in recent studies [1, 2, 8].

Thus, even in patients with thoracic trauma who do not have severe rib displacement, clinicians need to consider a delayed hemothorax when patients suddenly complain of prodromes, and should be thoroughly monitored to avoid missing the goldentime.

## AUTHORS’ CONTRIBUTIONS

Conceptualization: CHP. Data curation: KEK. Formal analysis: JWL. Funding acquisition: none. Methodology: CHP, KEK. Project administration: MCC, JWL. Visualization: CHP, MCC. Writing – original draft: CHP, KEK. Writing – review & editing: CHP, KEK,JWL.

## CONFLICT OF INTEREST STATEMENT

No potential conflict of interest relevant to this article was reported.
